# Web Health Monitoring Survey: A New Approach to Enhance the Effectiveness of Telemedicine Systems

**DOI:** 10.2196/resprot.5187

**Published:** 2016-06-06

**Authors:** Maria Francesca Romano, Maria Vittoria Sardella, Fabrizio Alboni

**Affiliations:** ^1^ Institute of Economics Scuola Superiore Sant'Anna Pisa Italy

**Keywords:** Web questionnaire, Web health monitoring survey, telemedicine, virtual checkup, survey quality, quality indicators, paradata

## Abstract

**Background:**

Aging of the European population and interest in a healthy population in western countries have contributed to an increase in the number of health surveys, where the role of survey design, data collection, and data analysis methodology is clear and recognized by the whole scientific community. Survey methodology has had to couple with the challenges deriving from data collection through information and communications technology (ICT). Telemedicine systems have not used patients as a source of information, often limiting them to collecting only biometric data. A more effective telemonitoring system would be able to collect objective and subjective data (biometric parameters and symptoms reported by the patients themselves), and to control the quality of subjective data collected: this goal be achieved only by using and merging competencies from both survey methodology and health research.

**Objective:**

The objective of our study was to propose new metrics to control the quality of data, along with the well-known indicators of survey methodology. Web questionnaires administered daily to a group of patients for an extended length of time are a Web health monitoring survey (WHMS) in a telemedicine system.

**Methods:**

We calculated indicators based on paradata collected during a WHMS study involving 12 patients, who signed in to the website daily for 2 months.

**Results:**

The patients’ involvement was very high: the patients’ response rate ranged between 1.00 and 0.82, with an outlier of 0.65. Item nonresponse rate was very low, ranging between 0.0% and 7.4%. We propose adherence to the chosen time to connect to the website as a measure of involvement and cooperation by the patients: the difference from the median time ranged between 11 and 24 minutes, demonstrating very good cooperation and involvement from all patients. To measure habituation to the questionnaire, we also compared nonresponse rates to the items between the first and the second month of the study, and found no significant difference. We computed the time to complete the questionnaire both as a measure of possible burden for patient, and to detect the risk of automatic responses. Neither of these hypothesis was confirmed, and differences in time to completion seemed to depend on health conditions. Focus groups with patients confirmed their appreciation for this “new” active role in a telemonitoring system.

**Conclusions:**

The main and innovative aspect of our proposal is the use of a Web questionnaire to virtually recreate a checkup visit, integrating subjective (patient’s information) with objective data (biometric information). Our results, although preliminary and if need of further study, appear promising in proposing more effective telemedicine systems. Survey methodology could have an effective role in this growing field of research and applications.

## Introduction

Survey methodology and medical research are becoming more connected: statisticians have always cooperated with clinicians in the analysis of collected data, and in recent years new research questions have arisen, along with new research fields (eg, health technology assessment and health economics). The aging European population and the interest in a healthy population in western countries have contributed to the increase in the number of health surveys, such as the English Longitudinal Study of Ageing (ELSA, since 2002) [1], the Survey of Health, Ageing and Retirement in Europe (SHARE, individuals from 20 European countries aged ≥50 years) [2], and the Health and Retirement Study (HRS) since 1992 in United States [3], to cite only the most well-known and relevant for the number of countries involved or for the systematic nature of the survey.

In all these surveys, as well as in more traditional clinical trials, the role of methodology in survey design, data collection, and data analysis is clear and recognized by the whole scientific community. The role of statisticians in clinical trials is now complemented by survey methodology: patients are often surveyed at the beginning and at the end of the trial to assess their well-being or other characteristics connected to the topic under investigation. Furthermore, patients are often requested to give feedback on their satisfaction with health structures, and, last but not least, health data management and analysis are now mandatory in health services assessment. Therefore, the contact points between medical and statistical research are numerous. Due to the complexity of research topics, the requested skills and knowledge are multidisciplinary and interdisciplinary: the role of researchers has been changing, and researchers now need to interconnect with each other, in order to achieve their research goals.

In the same years, survey methodology has had to couple with the challenges deriving from data collection through information and communications technology, the movement from landline telephones to mobiles and smartphones, and the increasing spread of Wi-Fi connections: modes and techniques to collect responses from people is always changing [4-8]. Guidelines and standards to follow are constantly being updated in order to be able to cover ever-changing and dynamic situations and data gathering modes [9,10], and new metrics derived from macro- and microparadata are being proposed and tested [11-16]. At the same time, the evolution of information and communications technology is giving a strong push to telemonitoring, telemedicine, and home rehabilitation systems [17-21]: new systems are being tested, and are quickly evolving from static to wireless devices, and from obtrusive to less-obtrusive equipment, also making it possible to follow patients and collect their information on their activities and reactions potentially everywhere and continuously.

In our opinion, there are new and common challenges for medicine and survey methodology. Which data are useful to be collected and analyzed? How? And when? What is the data’s value for the well-being of the patient, which is the ultimate aim of a telemonitoring system? What about the quality and usefulness of the data collected?

In the vast scientific literature on telemedicine systems, we have found only a few reports of systems that use data and information coming directly from patients [22-27], although several systems, as for example Philips’ eCareCompanion and eCareCoordinator [28], are now moving on to collecting this information through tablets or smartphones [29,30]. Surely researchers are exploring the implications of how and when these subjective data are collected, their utility for effective monitoring, and, more relevant from our point of view, whether there is a quality control process for this subjective information and how it works, but results have not reached the publication stage.

Our vision is that an effective telemonitoring system would be able to 1) collect objective and subjective data at the same time (biometric parameters and symptoms reported by the patients themselves), 2) control the quality of data collected, and 3) analyze data both at the individual and at an aggregated level.

This ambitious goal can be achieved only by using (and merging) all the technologies and skills available from both survey methodology and health research. In this paper we focus on the contribution of subjective data, that is, data reported by the patients themselves, about themselves, in way that simulates a standard medical examination: this, in our opinion, constitutes the real innovation in the scheme above.

### From Acquisition of Vital Signs to an Integrated Telemedicine System

It is quite obvious that remote monitoring and telemedicine systems apply only to patients with chronic conditions. For them, a remote monitoring system is extremely useful, as it is designed to store data collected directly at the patient’s home, with the goal of detecting as soon as possible any worsening of the patient’s health status.

Patient-doctor communication seems to be neglected in home monitoring, and yet it is always present in medical checkups (and in their telephone communications). Data and information are always the objects of the patient-doctor communication in each (first or follow-up) medical visit, and yet it seems that telemedicine systems do not include this relevant source of information. Generally, patient-doctor communication is face-to-face. In the first contact with the patient, the physician starts with a clinical interview: this allows the physician to know how the patient feels and the symptoms that he or she reports, and it precedes the objective physical examination. Thereafter, the patient undergoes further examinations (electrocardiography, echocardiography, chest x-ray, etc, and laboratory tests according to the physician’s request). Only after all the data and information have been collected and read is the physician able to confirm the diagnosis and prescribe suitable therapy.

We underline that—as physicians themselves state—listening to the patient’s account of his or her symptoms and feelings is key to acquiring all of the information about the patient’s health status. To have only biometric or laboratory data is not sufficient for making a correct diagnosis and consequent decisions, for taking actions, and for giving prescriptions. For patients with a chronic disease, there are recognized guidelines (standardized by each disease) that have to be followed in a medical checkup: guidelines recommend not only which laboratory and other tests to take, but also that the symptoms reported by the patients themselves be recorded. Last but not least, patients with chronic diseases are increasingly able to recognize and communicate correct and useful information about their conditions. From these considerations, it follows that a remote monitoring system must have the aim of daily collecting vital signs and biometric data, as well as subjective data, from the patient.

Information and communications technology tools and survey methodology allow for collection of these types of data according to the ideal standards: the questions (asked by a physician) and the responses (given by the patient) can be easily recognized as simulating a questionnaire, whose content and frequency of administration are determined by physicians.

### The Role of Survey Methodology in Health Monitoring

The medical checkup can be transformed into a computer-assisted Web interview, and the Web-collected data can be used to generate a longitudinal survey. Subjective data can be analyzed together with objective data in order to have an assessment of the patient’s health status or to detect potential risks.

In chronic diseases, the checkup visit can be more easily transformed into a structured questionnaire, because the clinical interview focuses mainly on presenting symptoms and their variations since the last visit. It is possible to acquire and store on a daily basis (or with a different periodicity; in any case, more frequently than the face-to-face checkup visits) all responses and information given by the patient. More important, it is possible to analyze both clinical and patient variables (measured at the same time) along appropriate longitudinal statistical models.

The aging of the population in western countries calls for the unavoidable and necessary use of a system to automatically assess the health status of patients with chronic conditions, supporting and complementing face-to-face clinic activities, with the aim of focusing the efforts of medical staff on the most serious cases. In order to reliably automate prediction of the state of health of a chronically ill patient, it is also necessary to ensure that data collection is at the highest possible level of reliability and that the data are validated according to metrics established and accepted by the scientific community.

To sum up, in a telemedicine system, questionnaires can be administered (almost) daily for a group of patients for an extended length of time. Are the questionnaires collected in a telemedicine system similar to a longitudinal survey? If we consider that a survey “is a systematic method for gathering information from (a sample of) entities for the purposes of constructing quantitative descriptors of the attributes of the larger population of which the entities are members” [5], then the answer is indeed positive, and we name it a Web health monitoring survey (WHMS).

### Quality Measures of a WHMS

If “the quality of a survey is best judged not by its size, scope, or prominence, but by how much attention is given to [preventing, measuring, and] dealing with the many important problems that can arise” [31], then it is relevant to compare the American Association of Public Opinion Research’s (AAPOR) criteria to “produce a quality survey” with the particular situation of a WHMS (Table 1) [31].

**Table 1:** Comparison between the American Association of Public Opinion Research’s (AAPOR) criteria [31] for a general survey and the features of a Web health monitoring survey (WHMS).

	AAPOR survey criteria	Features of a WHMS
Points better addressed by a WHMS	Have specific goals.	Have specific goals.
	Consider alternatives.	Consider alternatives.
	Take great care in matching question wording to the concepts being measured and the population studied.	Easier than in other fields of research. More attention to question wording in different languages or contexts.
	Maximize cooperation or response rates within the limits of ethical treatment of human subjects.	Patients are strictly involved and their active role in the monitoring system can stimulate a high participation rate, a high response rate, and a low item nonresponse rate. The patient AND the patient’s relatives or caregivers can have more positive feelings in participating in the survey.
Points easily respected by a WHMS	Select samples that well represent the population to be studied	The sample is not probabilistic, rather it resembles an opt-in one.
	Use designs that balance costs with errors.	Use designs that balance costs with errors.
	Pretest questionnaires and procedures.	Pretest questionnaires and procedures.
	Train interviewers carefully on interviewing techniques and the subject matter of the survey.	Train and supervise patients (and doctors).
	Use appropriate statistical analytic and reporting techniques.	Use appropriate statistical analytic and reporting techniques.
	Develop and fulfill pledges of confidentiality given to respondents.	Develop and fulfill pledges of confidentiality given to respondents.
	Disclose all methods of the survey to allow for evaluation and replication.	Disclose all methods of the survey to allow for evaluation and replication.
Point to be studied for a WHMS	Check quality at each stage.	New indicators and metrics are required.

## Methods

The main contribution that survey methodologists can make to WHMS is related to the quality of the survey at each stage.

As a first contribution, we offer some indicators, calculated on data collected during the trial of an integrated telemedicine system (*Assistenza domiciliare allo SCOmpenso cardiaco attraverso Le Tecniche Avanzate di comunicazione digitale*, or ASCOLTA, the Italian word for listen) [32] conducted in 2011 in Pisa, Italy, involving 12 patients with a diagnosis of heart failure. We collected both biometric and subjective data for a period of 2 months for 11 patients and for <1 month for 1 patient, who was the only one hospitalized for noncardiac problems and was thus not included in the study. Patients had to connect daily to a website, at the time most convenient for the patients themselves. A Web questionnaire—dynamically constructed with a different number of questions (and answers) according to the patient’s clinical condition—collected the patients’ health data, including additional physical data (weight and arterial pressures) measured and reported by the patients themselves. At the end of the questionnaire, the system asked the patient to wear wireless electrocardiograph, pulse oximetry oxygen saturation, and respiratory rate recorders for 5 minutes. The patients were free to ask for a new connection at their discretion.

We have previously described and discussed the positive contribution of subjective data to detecting potential risks [32]: variables obtained during a virtual visit substantially contributed to assessment of clinical status (69% of correct classifications), similar to the traditional biometric variables (70%), as assessed by a random forest classification algorithm. The combined use of both variables led to a more correct classification of the patient’s health status (84%).

The questionnaire (see Multimedia Appendix 1 for the English translation) was designed under cardiologists’ supervision, tested, and transformed into a Web questionnaire. The wording of questions and answers and their sequence were in accordance with European Society of Cardiology guidelines [33] for heart failure patients and with the cardiologists’ clinical experience.

We also collected paradata during the study period, along with instrumental and subjective data.

During the 2-month trial, we collected a total of 478 records from 11 patients, 243 for the first month and 235 for the second month.

As described before, a WHMS is a longitudinal survey that is quite different from a panel survey in terms of duration and contents; therefore, we needed more specific indicators than the well-known and consolidated indicators available in the literature [12,34,35].

We therefore propose the use of indicators that measure the response and attrition rates at the patient level. The item nonresponse rate is calculated as a proxy of the patient’s cooperation. To measure whether there was any habituation to a daily questionnaire, we compared the time span to complete the questionnaire between the two halves of the entire study period. Finally, we measured the consistency of the time chosen to complete the questionnaire as a proxy of the patient’s compliance and satisfaction. Focus groups with patients were organized and conducted both at the beginning and at the end of the trial.

## Results

### Patient Adherence and Response Rate

**Figure 1:**
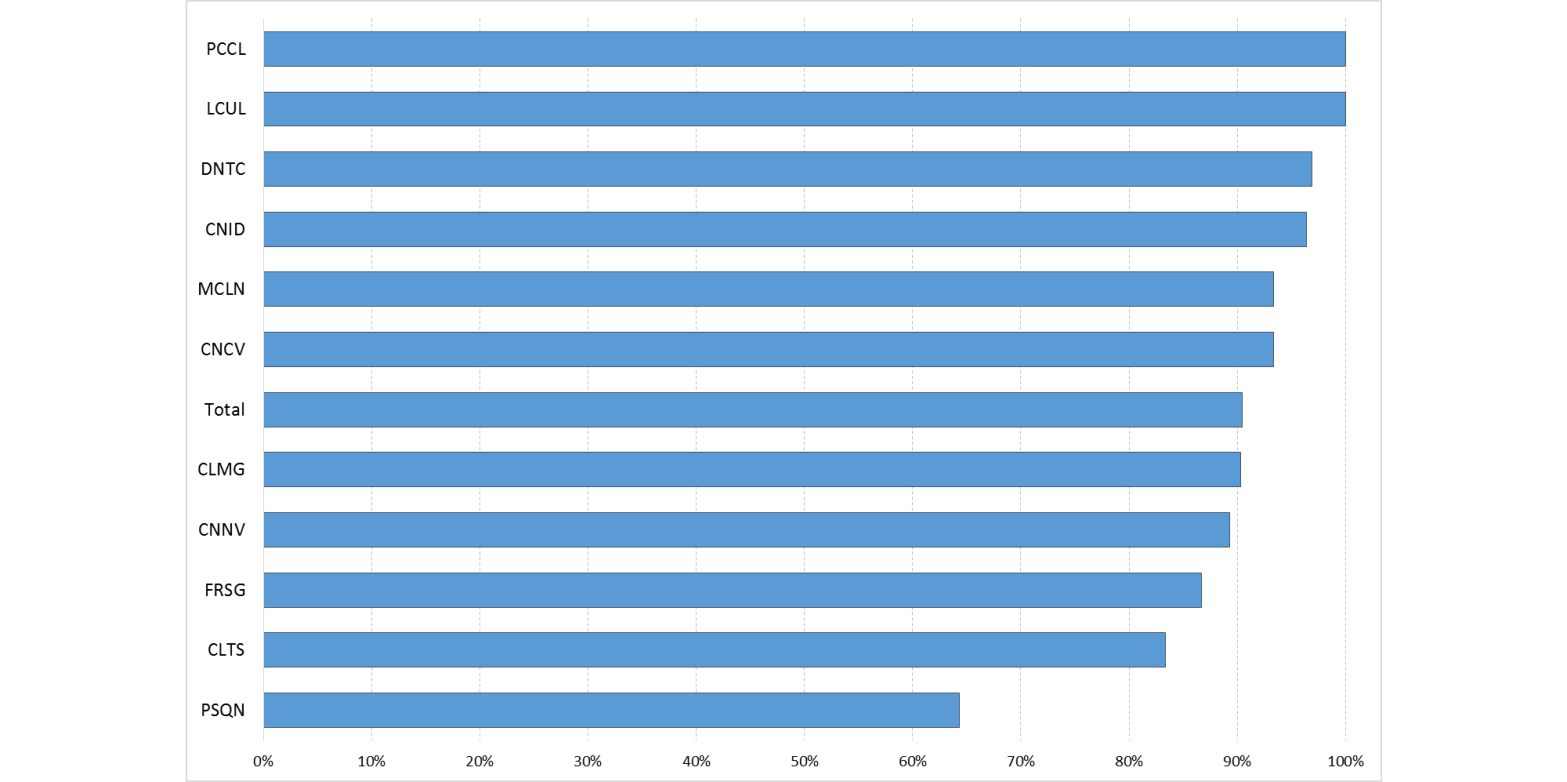
Response rate, by patient, during the 2-month ASCOLTA study.

As expected, patients were very cooperative due to an effective strategy to encourage patient involvement. The number of missing questionnaires was very low. Figure 1 illustrates the response rate by each patient.

### Item Nonresponse

We measured the rate of nonresponse to each item, obtaining good results (Table 2). The 3 biometric variables that the patients were asked to report in the questionnaire all had a nonresponse rate of 0%, which we interpret as denoting a cooperative attitude among all of the patients: during the final focus group the patients expressed positive reactions for their active role.

**Table 2:** Rate of nonresponse to questionnaire items (N=514).

Items	Nonresponses	
	n	%
Overall feeling	2	0.39
Weight relative to previous day	31	6.03
Swollen legs	5	0.97
Shortness of breath yesterday	4	0.78
Shortness of breath when combing	28	5.45
Shortness of breath when washing	34	6.61
Shortness of breath when getting dressed	38	7.39
Shortness of breath when tying shoes	31	6.03
Shortness of breath when climbing steps	33	6.42
Palpitations	2	0.39
Chest tightness	2	0.39
Tiredness	2	0.39
Maximum blood pressure	0	0
Minimum blood pressure	0	0
Weight	0	0

**Figure 2:**
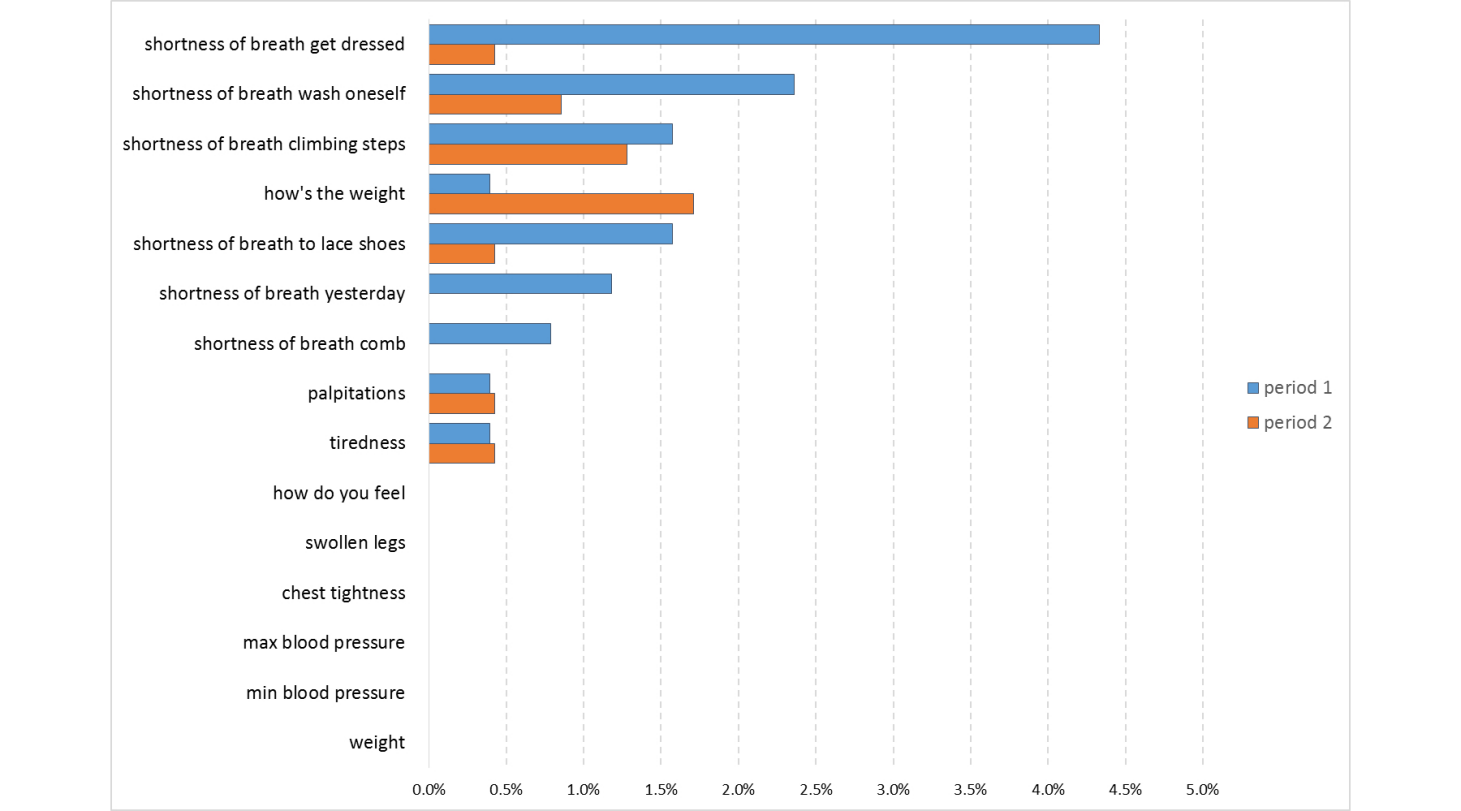
Comparison of nonresponses to questionnaire items by period of the study (period 1: first month; period 2: second month) and by question.

To measure habituation to the questionnaire, we compared the item nonresponses between the first and the second month. We found no significant differences (Figure 2).

### Time to Complete the Questionnaire

From paradata collected, we calculated the time spent to complete the questionnaires both for the daily and the discretionary questionnaires (Table 3). The time required seemed to depend on the patients’ health conditions; in general, time required was very short and acceptable, and at our advice was not perceived as a burden by the patient.

**Table 3:** Time spent to complete the daily and discretionary questionnaires, by patient.

Patient	Daily		Discretionary		Total	
	n	Mean time (min.s)	n	Mean time (min.s)	n	Mean time (min.s)
CLMG	73	1.07	1	8.00	74	1.12
CLTS	55	1.08	0	0	55	1.08
CNCV	20	1.36	1	4.00	21	1.43
CNID	85	1.46	1	1.00	86	1.45
CNNV	24	1.03	1	1.00	25	1.02
DNTC	22	3.49	3	2.20	25	3.38
FRSG	27	1.27	1	1.00	28	1.26
LCUL	46	1.46	0	0	46	1.46
MCLN	57	1.09	1	1.00	58	1.09
PCCL	50	1.34	5	1.24	55	1.33
PSQN	19	1.16	2	1.30	21	1.17
Total	478	1.31	16	2.04	494	1.32

**Figure 3:**
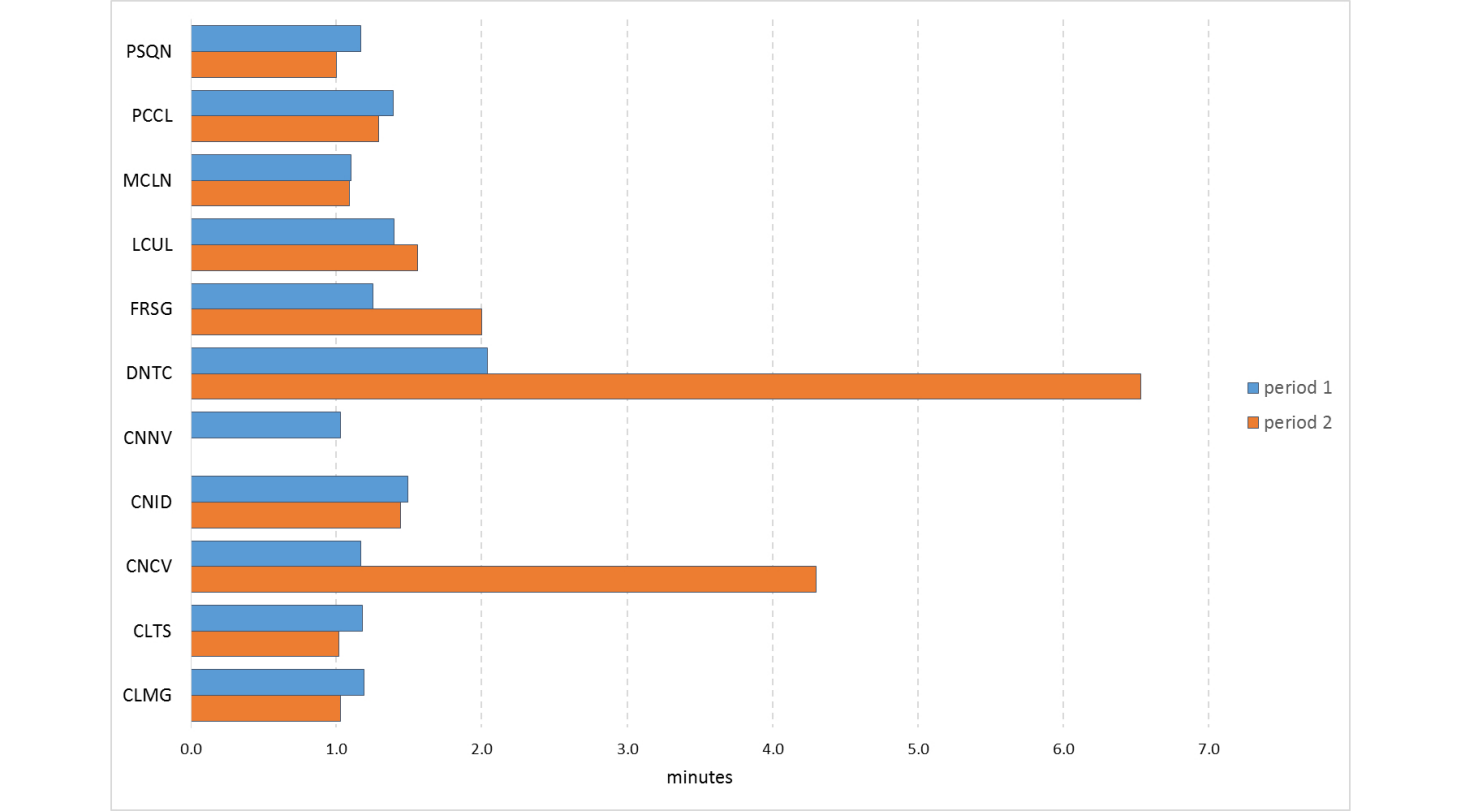
Time spent to complete the questionnaires, by patient and by period of the study (period 1: first month; period 2: second month).

We also tested for difference in completion time between the first and second period (Figure 3 and Table 4). Although the difference was statistically significant for 3 patients, the overall difference was not.

**Table 4:** Time (min.s) spent to complete the daily questionnaires: comparison between first and second period of the study, by patient.

Patient	Period		*t* test	*P* value	*df*
	First half	Second half			
CLMG	1.09	1.05	0.71	.48	71
CLTS	1.16	1.02	2.10	.04^a^	53
CNCV	1.00	2.20	–2.47	.02^a^	18
CNID	1.37	1.56	–0.51	.61	83
CNNV	1.05	1.00	0.92	.37	22
DNTC	2.04	6.53	–1.71	.10	20
FRSG	1.47	1.05	1.18	.25	25
LCUL	1.37	2.10	–1.10	.28	44
MCLN	1.08	1.14	–0.49	.63	55
PCCL	1.30	1.41	–0.49	.63	48
PSQN	1.30	1.05	2.13	.05^a^	17
Total	1.25	1.37	–1.06	.29	476

^a^Significant difference (*P* ≤.05).

### Patients’ Chosen Time of Day to Complete the Questionnaire

To accommodate the patient’s lifestyle, each one could choose the hour of the day when they would complete the questionnaire, with an interval of ±1 hour (Table 5).

During the final focus group, the patients expressed their appreciation for this support, and therefore their behavior is a proxy of a cooperative attitude.

**Table 5:** Time of day chosen to complete the questionnaire, by patient.

Patient	Total no. of daily questionnaires completed	Median time at start of questionnaire (h.min.s)	Mean time at start of questionnaire (h.min.s)	Mean difference from median	Mean difference from mean
CLMG	73	8.05.00	8.13.39	18.27	17.54
CLTS	55	14.42.00	14.42.11	16.13	15.46
CNCV	20	15.23.00	15.33.14	13.53	15.51
CNID	85	17.48.30	17.45.53	14.28	14.42
CNNV	24	19.24.30	19.14.28	24.33	26.3
DNTC	22	9.45.00	9.55.55	22.55	25.3
FRSG	27	9.29.30	9.25.56	18.13	18.11
LCUL	46	8.20.00	8.26.29	22.26	22.12
MCLN	57	9.20.00	9.30.47	17.02	19.25
PCCL	50	7.56.30	8.36.28	11.51	31.42
PSQN	19	20.07.00	19.58.11	20.09	19.32

The mean difference was about 20 minutes, both from the median and from the mean.

## Discussion

It is worth noting that our WHMS is part of a telemedicine system, making its goal different from that of other health surveys such as a Web-based daily questionnaire for health [26]. Telemedicine data are collected and must be analyzed at the patient level, because the main goal is to assess the health status of the single patient. On the contrary, in epidemiological studies the focus is on measuring health parameters for the entire population.

Moreover, a WHMS is designed for patients with chronic conditions, and therefore they may already be greatly involved in the survey or their cooperative attitude can be reinforced: no incentive is needed, because the implicit incentive for each patient is to get better attention from the medical staff and to contribute actively to the management of their chronic disease. As a consequence, all indicators based on response rate (although—or perhaps because—they have values never obtained in a general survey) seem less relevant to measure the quality of the WMHS. Nonetheless, their trend over time for longer study is worth computing and controlling.

WHMS is a longitudinal survey with very strict periodicity, even daily, which could be a burden for the respondent [36], resulting in a possible high attrition rate. The attrition rate at the patient level is therefore relevant in monitoring the quality of data collection. Our results demonstrate that this risk is very low, taking into account that only a few minutes are required to complete the questionnaire.

Even with a diligent patient, there is the risk that patients’ responses will become automatic. The results we obtained led us to reject the hypothesis of the presence of automatic responses; therefore, the patient’s responses are deemed to be reliable.

Signing in to the daily questionnaire and the patients’ choice of when during the day to sign in seem a good measure of their cooperation.

Focus groups with patients confirmed the efficacy of the questionnaire and the positive reactions of patients to the new mode of collecting this information. Patients appreciated their new active role in the ASCOLTA system.

### Study Limitations

We acknowledge that our study has some critical limitations, such as the small number of patients studied and the short duration of the longitudinal survey. Furthermore, the study involved only patients with heart failure. Other paradata can be collected, such as those needed to measure response latency or time-to-click [16,37].

In more general terms, the digital skills of potential respondents to a WHMS is a challenge: a sample of patients with chronic conditions would be older, and the greater digital divide in this group could be a barrier to their participation in such a survey [38-40].

Many aspects of a WHMS deserve to be developed, by applying the same approach to other kinds of chronic pathologies (eg, chronic obstructive pulmonary disease, diabetes, and hypertension) [41,42]. Moreover, the expansion of wireless technologies on platforms such as smartphones and tablets will enable the collection of biometric data to extend even to people who are not yet ill (eg, people at risk) or to healthy people who participate in amateur sports. In addition, although this is an open field, the reactions of medical staff to the virtual visit are worth investigating, as patient satisfaction is further explored [43-47].

Information and communications technology solutions can be applied to facilitate and enable patient-doctor communication [48-53]. Many virtual medical visit applications are being developed [54-56] and the increasing number of websites testifies to patients’ willingness to play a more active role in their own health management [57,58].

The main and innovative aspect of our proposal is the use of a Web questionnaire to virtually recreate a checkup visit, integrating subjective (patient information) with objective data (biometric information) in a unique database that can be analyzed with appropriate statistical methods. We suggest here some indicators to control the process of data collection, along with criteria already established by survey methodology, but we are conscious that other specific metrics need to be studied.

Our results, although preliminary, appear promising and, in our opinion, could be of significance to the ongoing debate about the most appropriate type of telemonitoring and remote care of patients. Further studies, in a larger population, could be useful to ultimately confirm our preliminary results and to provide a more efficient cost-benefit ratio, according to health technology assessment.

Survey methodology could have an effective role in this growing field of research and applications.

In agreement with the Couper’s statement that “We constantly need to hone our skills, update our knowledge, and expose ourselves to new developments in other disciplines and fields of research and application.” [59], we think that monitoring of health data is a challenge for survey methodologists. Skills and competencies of survey methodologists and statisticians must guide data collection and data analysis in health monitoring surveys.

## Appendix

Multimedia Appendix 1 Questionnaires used (English translation).

## Abbreviations

AAPOR: American Association of Public Opinion Research
ASCOLTA: Assistenza domiciliare allo SCOmpenso cardiaco attraverso Le Tecniche Avanzate di comunicazione digitale
ELSA: English Longitudinal Study of Ageing
HRS: Health and Retirement Study
SHARE: Survey of Health, Ageing and Retirement in Europe
WHMS: Web health monitoring survey
