# Supplementary material for: Web Health Monitoring Survey: A New Approach to Enhance the Effectiveness of Telemedicine Systems
Source: JMIR Res Protoc. 2016 Jun 6;5(2):e101. doi: 10.2196/resprot.5187 (PMC4914780; doi:10.2196/resprot.5187)
Supplement: Multimedia Appendix 1 [file resprot_v5i2e101_app1.pdf]

Daily Questionnaire

The following questions, which you have to answer once a day, are useful to check your condition.
Answer following the instructions. Thank you.
Also in accordance with Legislative Decree 196/03 on privacy, we assure you that your answers will not be made public.

How do you feel? *(select only one response)*
□ well □ not too well □ bad

How is the weight compared to yesterday? *(select only one response)*
□ stable □ increased □ decreased

Please write the number on your scale: _________

Measure the blood pressure with the tool provided and write
the minimum pressure ____
the maximum pressure ____

Are your legs swollen? *(select only one response)*
□ they are not swollen □ they were swollen last night □ they were always swollen

Were you able to put your shoes on? *(select only one response)*
□ Yes □ No
Have you followed correctly the diuretic therapy? *(select only one response)*
□ Yes □ No

How's the shortness of breath with reference to yesterday? *(select only one response)*
□ it has improved □ it is stable □ it has worsened

How was the shortness of breath the last time you: *(for each of the following actions choose a number from 1= very mild to 5 = severe)*

**combed**	very mild 1 2 3 4 5 severe
**washed**	very mild 1 2 3 4 5 severe
**dressed**	very mild 1 2 3 4 5 severe
**tied your shoes**	very mild 1 2 3 4 5 severe
**climbed at least 3 steps**	very mild 1 2 3 4 5 severe

Have you experienced palpitations in the last 24 hours? *(select only one response)*
□ Yes □ No

Have you had tightness in the chest in the last 24 hours? *(select only one response)*
□ Yes □ No

connected to physical efforts? *(select only one response)*
□ Yes □ No
and at rest? *(select only one response)*
□ Yes □ No

How much do you feel tired at the moment? *(choose a number from 1 = not tired to 5 = very tired)*
Not tired 1 2 3 4 5 very tired

Did you fill the questionnaire:: □ by myself □ with the help of other people

The questions are over, thanks for your answers. You will need to repeat this questionnaire tomorrow.
You have to make an instrumental analysis of few minutes.
Connect the sensors, turn on the device and wait about 2 minutes.

At request questionnaire

Hello Mr / Mrs XY.
The next analysis is planned for the day xx at yy hour.
In the meantime, if you wish, you can perform further analysis. In this case, press the "start" button.

Why do you want to repeat the exam? Please write what happened and what you're afraid of:
____

How do you feel now? *(select only one response)*
□ well □ not too well □ bad

How is the weight compared to yesterday? *(select only one response)*
□ stable □ increased □ decreased

Measure the blood pressure with the tool provided and write
the minimum pressure ____
the maximum pressure ____

Are your legs swollen? *(select only one response)*
□ they are not swollen □ they were swollen last night □ they were always swollen

Were you able to put your shoes on? *(select only one response)*
□ Yes □ No
Have you followed correctly the diuretic therapy? *(select only one response)*
□ Yes □ No

How's the shortness of breath compared to the previous link? *(select only one response)*
□ it has improved □ it is stable □ it has worsened

Have you had palpitations since the previous link? *(select only one response)*
□ Yes □ No

Have you had tightness in the chest since the previous link? *(select only one response)*
□ Yes □ No

connected to physical efforts? *(select only one response)*
□ Yes □ No
and at rest? *(select only one response)*
□ Yes □ No

How much do you feel tired at the moment? *(responds by choosing a number from 1 = not tired to 5 = very tired)*
Not tired 1 2 3 4 5 very tired

Did you fill the questionnaire:: □ by myself □ with the help of other people

You have to make an instrumental analysis of few minutes.
Connect the sensors, turn on the device and wait about 2 minutes.
